# Long term disease-free survival and T cell and antibody responses in women with high-risk Her2+ breast cancer following vaccination against Her2

**DOI:** 10.1186/1479-5876-5-42

**Published:** 2007-09-06

**Authors:** Michael A Morse, Amy Hobeika, Takuya Osada, Donna Niedzwiecki, Paul Kelly Marcom, Kimberly L Blackwell, Carey Anders, Gayathri R Devi, H Kim Lyerly, Timothy M Clay

**Affiliations:** 1 Department of Medicine, Division of Medical Oncology, Duke University Medical Center, Box 3233, Durham, NC 27710, USA; 2 Department of Surgery, Duke University Medical Center, Box 2606, Durham, NC 27710, USA; 3 Department of Biostatistics & Bioinformatics, Duke University Medical Center Box 3958, Durham, NC 27710, USA; 4 Department of Medicine, Division of Medical Oncology, Duke University Medical Center, Box 3147, Durham, NC 27710, USA; 5 Department of Medicine, Division of Medical Oncology, Duke University Medical Center, Box 3893, Durham, NC 27710, USA; 6 Duke Comprehensive Cancer Center (DCCC), Duke University Medical Center, 6^th ^Floor Hock Plaza, Durham, NC 27710, USA; 7 Department of Surgery, Division of Experimental Surgery, Duke University Medical Center, Box 2606, Durham, NC 27710, USA

## Abstract

**Background:**

The HER2-inhibiting antibody trastuzumab, in combination with chemotherapy, significantly improves survival of women with resected, HER2-overexpressing breast cancers, but is associated with toxicities including a risk of cardiomyopathy. Additionally, the beneficial effect of trastuzumab is expected to decrease once the drug is discontinued. We proposed to address these concerns by using cancer vaccines to stimulate HER2 intracellular domain (ICD)-specific T cell and antibody responses.

**Methods:**

Subjects with stage II (≥ 6 +LN), III, or stage IV breast cancerwith > 50% HER2 overexpressing tumor cells who were disease-free after surgery and adjuvant therapy were eligible. Vaccines consisted of immature, cultured DC (n = 3), mature cultured DC (n = 3), or mature Flt3-ligand mobilized peripheral blood DC (n = 1) loaded with ICD, or tetanus toxoid, keyhole limpet hemocyanin or CMV peptide as controls, and were administered intradermally/subcutaneously four times at 3 week intervals. ICD-specific T cell and antibody responses were measured. Cardiac function was determined by MUGA or ECHO; long term disease status was obtained from patient contact.

**Results:**

All seven patients successfully underwent DC generation and five received all 4 immunizations. There were no toxicities greater than grade 1 or ejection fraction decrements below normal. Delayed-type hypersensitivity (DTH) reactions at the injection site occurred in 6/7 patients and HER2 specificity was detected by cytokine flow cytometry or ELISPOT in 5 patients. At more than 5 years of follow-up, 6/7 had detectable anti-ICD antibodies. One patient experienced a pulmonary recurrence at 4 years from their study immunizations. This recurrence was resected and they are without evidence of disease. All patients are alive and disease-free at 4.6–6.7 years of follow-up.

**Conclusion:**

Although this was a small pilot study, the well-tolerated nature of the vaccines, the lack of cardiac toxicity, significant immunogenicity, and a 100% 4.5-year survival rate suggest that vaccination with HER2 ICD protein-containing DC is appropriate for further study in this population.

**Trial Registration:**

ClinicalTrials.gov NCT00005956

## Background

HER2 overexpression occurs in 20–30% of breast cancers and is associated with more aggressive tumors and poorer overall survival (OS) in those with resected disease [1]. Recently, the benefit of combining the anti-HER2 antibody trastuzumab with chemotherapy in reducing the rate of recurrence mortality of resected, HER2 overexpressing breast cancer was established [2]. Although the addition of trastuzumab significantly improved survival in these studies, it has been associated with toxicities including cardiac dysfunction and, less frequently, interstitial pneumonitis. Furthermore, the effect of trastuzumab is only expected to persist while it remains at clinically relevant concentrations. For these reasons, we sought to study the role of an alternative strategy to target the intracellular domain (ICD) of HER2 via stimulation of HER2-specific T cell and antibody responses using cancer vaccines.

More than a dozen phase I and phase II studies have been conducted in breast cancer patients with cancer vaccines [3], that have included proteins, peptides, modified tumor cells, and dendritic cells loaded with breast tumor antigens. In these studies, HER2 has been demonstrated to be immunogenic [4-13]. For example, in a study of 31 patients with stage III or IV HER2+ breast cancer who received 6 monthly vaccinations consisting of a T helper epitope from HER2 protein plus GM-CSF, 92% of the patients demonstrated HER2 immunity as measured by T cell proliferation. Importantly, immunity lasted for at least 1 year in 38% of responding patients [7]. Recently, a vaccine consisting of a peptide derived from the extracellular domain of HER2 (E75 peptide (HER2 369–377)) mixed with GM-CSF was administered at various doses and schedules to patients with resected node positive and node negative breast cancer. All patients demonstrated in vivo DTH responses and in vitro immunologic responses following vaccination. The recurrence rate for the vaccinated patients was 5.6% compared to 14.8% for an observational group of patients at a median of 24 months [12,13].

To improve upon the immunologic and clinical activity of HER2-directed vaccines, we chose dendritic cells (DC) as the platform for delivering HER2 antigen. DC are the most effective antigen presenting cell for activating CD8+ cytolytic T cells, CD4+ T cell help, and antibody responses [14]. Furthermore, most cancer vaccines require cross presentation of the administered antigen by DC [15]. We therefore hypothesized that vaccines based on DC loaded with tumor antigens would provide potent antitumor responses with low toxicity, precise specificity, and a sustained effect (due to immunologic memory). There are several possible sources of DC for immunotherapy strategies [16]; DC may be generated in vitro from monocytoid precursors or CD34+ progenitors [17]. DC may also be found circulating in the peripheral blood and their numbers may be markedly enhanced after administration of Flt3-ligand [18]. Because there is a debate over whether mature or immature DC are the preferred cell source for cancer vaccine strategies, we designed our study as a series of pilot experiments with one group of patients receiving immature DC, then the next mature DC, and finally, Flt3-ligand mobilized DC. Because the efficacy of immunotherapy may be greatest in the setting of low burden of tumor when tumor-induced immune suppression is less likely, we tested DC-based immunization strategies in women with resected HER2-expressing breast cancer with a high risk of recurrence. We previously reported preliminary data for the immunization response in a subset of these patients [17]. We now report on long-term safety and survival of the entire group of women who received DC loaded with HER2 ICD protein fragment as vaccine therapy for high-risk breast cancer. In addition, we report on antibody responses detectable after more than 5 years of follow-up.

## Methods

This phase I study was reviewed and approved by the Duke University Institutional Review Board in accordance with all federal, state and local requirements. Patients provided written informed consent prior to receiving any protocol therapy.

### Patients

Patients were eligible if they had invasive (defined as stage II with at least 6 positive lymph nodes, stage III, or resected stage IV) HER2-overexpressing (2+ or 3+ by immunohistochemistry (IHC) or FISH amplified) breast cancer, a group with a historically high (30–50% or greater) risk of recurrence. In addition to having undergone a complete surgical resection, they were to have completed adjuvant therapy consisting of chemotherapy (± trastuzumab) and/or radiotherapy at least 4 weeks prior to study entry, have normal cardiac function, no evidence of autoimmune disease, and be free of corticosteroids. Continuation of adjuvant hormonal therapy was permitted.

### Dendritic Cell Vaccine

Patients underwent a 2–4 hour leukapheresis in order to obtain peripheral blood mononuclear cells (PBMCs) for generation of DC. Three-quarters of the pheresed PBMCs were utilized for DC and one-quarter was frozen for baseline immunologic study. The first DC product for each patient was generated and used fresh and the remainder were cryopreserved and thawed when needed for subsequent immunizations. The DC were generated *ex vivo *in one of three ways: The first three patients (Her2-I-01, Her2-I-02, and Her2-I-03) were immunized with immature DC generated from their PBMCs with GM-CSF and IL-4 over seven days, and loaded with the HER2 ICD protein (500 μg) (generously provided by Corixa, Inc., Seattle, WA). Control immunizations consisted of similarly prepared DC loaded with tetanus toxoid (TT) 4 Lf and keyhole limpet hemocyanin (KLH) 200 μg. The next three patients (DC-ICD-MAT-01, DC-ICD-MAT-02, DC-ICD-MAT-03) were immunized with DC generated from their PBMCs with GM-CSF and IL-4, loaded with the ICD of HER2 protein, the E75 extracellular domain (ECD) peptide, and then matured with CD40-ligand plus IFNγ. Control immunizations consisted of similarly prepared DC loaded with keyhole limpet hemocyanin (KLH) 200 μg, or CMV pp65 peptide 500 μg. The third cohort was to utilize blood DC mobilized with FLT3-ligand, loaded with ICD protein overnight and then matured with GM-CSF, CD40-ligand and IFNγ. Control immunizations consisted of similarly prepared DC loaded with tetanus toxoid (TT) 4 Lf, keyhole limpet hemocyanin (KLH) 200 μg, and CMV pp65 peptide 500 μg. A single patient (FL-DC-MAT-01) received that preparation, because at that point the ICD protein and FLT3-ligand became unavailable. Consequently, no further patients were enrolled on this trial. A total of 4 injections were to be given every 3 weeks as a combination of intradermal (100 microliters) and subcutaneous (900 microliters) injections. Patients were followed clinically with routine imaging requested approximately every 3 months to confirm patient-reported disease status. MUGA scans or echocardiograms were recommended upon completion of the immunizations to monitor ejection fraction.

### Immunologic analysis

#### DTH reactions

The clinical method for measuring immunologic immune responses consisted of measuring the longest diameter of induration and erythema at the injection site 48 hours after each immunization (reported as the delayed type hypersensitivity (DTH) reaction). In addition, prior to each immunization and at completion of the series of immunizations, patients received intradermal injections of 10 mcg of ICD protein or Candida at 1:1000 dilution (0.1 ml) to assess reactivity to the immunizing antigen compared with a control. Patients who received DC loaded with KLH also had DTH testing with 10 mcg KLH. The maximum diameter of erythema and induration was measured at 48 hours after the intradermal injection.

#### ELISpot

Peripheral blood mononuclear cells were analyzed for immunologic analysis as follows: Immulon-2 96-well flat bottom plates were coated with 50 μL/well antihuman interferon-γ (IFN-γ) monoclonal antibody (MoAb; M700A, Endogen, Perbio Science, HelsingboFrg, Sweden) in a coating buffer (1:500) and incubated for a minimum of 2 hours at room temperature or overnight at 4°C. The coated plates were washed 10 times with phosphate buffered saline (PBS), 0.1% Tween 20, then blocked with fetal bovine serum (FBS)-containing medium for 30–60 minutes at 37°C, 5% CO_2_. Effector cells were plated in quadruplicate in serial dilutions starting with 2 × 10^5 ^cells/well. Antigen was added to the wells and the plates were incubated for 48–50 hours at 37°C, 5% CO_2_, and then washed. Antihuman IFN-γ biotinylated MoAb 50 μL/well at 1:500 in 1% FBS/PBS was added and incubated at room temperature for 2 hours or 4°C overnight, and then washed. Alkaline phosphatase-strepavidin 50 μL/well (1:200) in 1% FBS/PBS was added and incubated at room temperature for 2 hours, and then washed. BCIP/NBT (5-bromo-4-chloro-3'-indolphosphate p-toluidine salt/nitro-blue tetrazolium chloride) substrate (Vector Laboratories, Burlingame, CA) 50 μL/well was added and incubated for 1 hour at room temperature in the dark, and then rinsed 3 times with deionized water. The membranes were dried overnight in the dark. Membranes were removed using sealing tape (Millipore, Bedford, MA) and were read by Zellnet Consulting, Inc, using the KS ELISA Automated Reader System with the KS ELISA 4.2 software (Carl Zeiss, Inc, Thornwood, NY).

#### Cytokine Flow Cytometry

Intracellular cytokine staining was performed by incubating 2–4 × 10^6 ^PBMCs isolated pre-immunization and post immunizations for 5 hours at 37°C in the presence of anti-CD28 and media alone, ICD protein, KLH protein, and TT (Sigma-Aldrich, St. Louis, MO). Brefeldin A was added for the last 3 hours of incubation. The stimulated cells were washed with 1% bovine serum albumen/PBS, fixed with 1% paraformaldehyde, and permeabilized using FACS™ Permeabilizing Solution (Becton Dickinson, San Jose, CA). Permeabilized cells were then stained with anti-IFN-γ-FITC, anti-CD69-PE, anti-CD8-PerCP, and anti-CD4-APC for 30 minutes at room temperature. Cells were gated by forward and side scatter for lymphocytes and positively for CD8. CD69 is an activation marker. The percentage of CD69+/IFN-γ+ cells (ie, activated and cytokine producing) was then determined.

#### HER2-ICD ELISA

HER2-ICD protein was dissolved in PBS (10 μg/ml). High binding 96-well ELISA plates (Immulon 4HBX; ThermoLabsystems, Franklin, MA) were coated with HER2-ICD solution (200 μl/well) for overnight at 4°C. After washing with 0.05% Tween20 in PBS (PBS-T), 200 μl of 1% bovine serum albumin in PBS was added for blocking of the wells and incubated for 2 hours at room temperature. Serially diluted patients' sera (200 μl/well) were added to the plates and incubated overnight at 4°C. Control serum from a normal donor was used as a negative control. Washed with PBS-T, and alkaline phosphatase-conjugated goat anti-human IgG (MP Biomedicals, Inc.; Aurora, Ohio) was put to the plates (1:5000 dilution in PBS) and incubated for 3 hours at 37°C. Unbound conjugate was removed by washing with PBS-T, and 1 mg/ml solution of p-nitrophenyl phosphate in Tris buffer (Sigma, St. Louis, MO) was added to the wells (50 μl/well). Color development was stopped by adding 50 μl of 3N NaOH. Absorbance at 405 nm was measured.

### Statistical analysis

Results were immunologic analyses were primarily descriptive. A Kaplan-Meier disease-free survival estimate and 95% confidence bounds was generated for the clinical data.

## Results

### Patient Characteristics

Seven women with a median age of 47 were enrolled onto the study (Table 1). Five patients had stage III and two had stage IV breast cancer. The median number of positive lymph nodes was 7 (range: 0–11). HER2 expression by IHC was high in all patients; specifically, 100% of the tumor cells in two patients, 80% in one patient, and all others were reported by the pathologist to be ≥ 2+. Two patients had ER/PR negative tumors. Prior therapy included high-dose chemotherapy with peripheral blood stem cell transplantation, hormonal therapy, chemotherapy, and post-surgical radiotherapy, and in one case, a dendritic cell vaccine loaded with CEA mRNA. Two patients had received trastuzumab, one for 12 weeks and the other for 1 year prior to enrollment. Three of the women were receiving maintenance hormonal therapy.

**Table 1:** Patient characteristics (n = 7)

Patient	Age	Disease Stage	Number + Lymph Nodes	HER2 Status	Prior Treatment/s	Maintenance Hormones
**Vaccinations: immature DC (GM-CSF, IL-4) + HER2 ICD protein control DC loaded with KLH and tetanus toxoid**						

#1	38	T3pN3 – IIIC	> 10	> 80% cells overexpress by IHC	AC-Taxol; trastuzumab	Raloxifene (ER/PR+)
#2	56	T1cpN2M1 – IV	7	100% cells overexpress by IHC	Taxol; HDC + Auto PBSCT DC-CEA vaccine	Tamoxifen (ER/PR+)
#3	57	T3pN2 – IIIC	7	3+	doxorubicin HDC + Auto PBSCT	None (ER/PR-)

**Vaccinations: Mature DC (GM-CSF, IL-4, CD40-ligand, IFNγ) + HER2 ICD protein, E75 ECD peptide control DC loaded with KLH and CMV peptide**						

#4	47	T1pN2 – IIIA	8	3+	AC; Taxotere; Radiotherapy	None (ER/PR-)
#5	58	T2pN3 – IIIC	11	2+	FAC; Taxol; XRT	Tamoxifen (ER/PR+)
#6	38	IV (liver)	0	High	Taxotere; Trastuzumab (prior to enrollment and then continued on maintenance > 4 years); FAC; Navelbine; RFA of liver lesion	None (ER/PR-)

**Vaccinations: DC (FLT3-ligand) + GM-CSF + CD40-ligand + IFNγ+ HER2 ICD protein control DC loaded with KLH, tetanus toxoid and CMV peptide**						

#7	45	T4N1 vs N0M0 – IIIB	0 @ surgery	3+ in 100% cells by IHC	Neoadj AC; post-op Taxol; post-mastect RT	None (ER/PR-)

F = Fluorouracil, A = doxorubicin, cyclophosphamide; HDC + PBSC = high dose chemotherapy followed by peripheral blood stem cell transplant; RFA = radiofrequency ablation;

IHC = immunohistochemistry

Note: Different IHC methodologies were used by pathologists in characterizing HER2 expression during the time patients were enrolled in the study. FISH was not utilized.

All patients successfully underwent leukapheresis and generation of at least one DC product. Viability of cryopreserved DC was 70–98%. The immature DC had the expected phenotype (CD83 low, CD86+HLA-DR+) and the matured DC expressed upregulated CD83 (range: 16–98% CD83+) among both fresh and cryopreserved DC. The matured DC preparations demonstrated IL-12 production. Specifically, within the DC of three patients analyzed (two in the cohort with monocyte derived and matured DC and the patient receiving Flt3-ligand mobilized and matured DC), intracellular IL-12 expression was demonstrated in 2–8% of the CD83+ cells, indicating an activated state.

### Immune Response

We used as the primary indicator of immune response DTH reactions observed at the vaccine injection sites (Table 2). These DTH reactions to the ICD loaded vaccine (erythema and/or induration), ranging from no reaction up to105 mm in longest diameter, were observed at the injection site in all but the one patient who received only a single immunization with the DC. In four patients, the diameter of the induration/erythema increased from the first to a subsequent immunization, and in two patients the first immunization gave the greatest induration/erythema. We also examined reactivity to the antigens administered intradermally without dendritic cells before and after all immunizations. Among five patients who were analyzed, two had an increase in size in the diameter of erythema/induration at the intradermal injection site following the immunizations, one had a decrease in size, and two patients had no DTH reactivity to ICD. When responses to Candida were similarly analyzed, only one of five had an increase in the diameter of the DTH reaction, while four patients had a decrease following all the immunizations. This suggests that responses against the HER2 were likely specific and not due to a non-specific increase in DTH reactivity.

**Table 2:** Longest DTH diameter at injection site of DC loaded with various antigens

**Patient**	**Longest diameter of induration at site of DC loaded with ICD (mm)**	**Longest diameter of erythema at site of DC loaded with ICD (mm)**	**Longest diameter of induration at site of DC loaded with TT (mm)**	**Longest diameter of erythema at site of DC loaded with TT (mm)**	**Longest diameter of induration at site of DC loaded with CMV (mm)**	**Longest diameter of erythema at site of DC loaded with CMV (mm)**	**Longest diameter of induration at site of DC loaded with KLH (mm)**	**Longest diameter of erythema at site of DC loaded with KLH (mm)**
#1	63 mm	64 mm	39 mm	50 mm	NA	NA	6 mm	18 mm
#2	35 mm	36 mm	20 mm	25 mm	NA	NA	20 mm	30 mm
#3	65 mm	105 mm	70 mm	70 mm	NA	NA	55 mm	135 mm
#4	25 mm	80 mm	45 mm	90 mm	30 mm	80 mm	65 mm	120 mm
#5	20 mm	30 mm	NA	NA	NR	20 mm	NR	10 mm
#6	20 mm	40 mm	NA	NA	10 mm	20 mm	10 mm	20 mm
#7	NR	NR	NA	NA	NR	NR	NR	NR

NR = No reaction at the injection site

NA = Not available (i.e., immunization not performed with the antigen listed)

**Figure 1:**
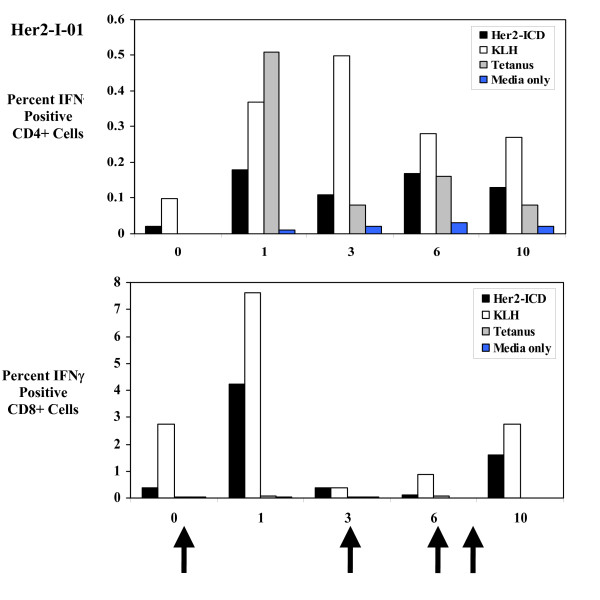
**Intracellular IFNγ production in CD4+ and CD8+ cells in response to HER2-ICD**. PBMC from patient Her2-I-01 were stimulated with HER2-ICD (50 μg/ml), KLH (50 μg/ml), Tetanus toxoid (5 Lf/ml) or media alone and analyzed for intracellular IFNγ at weeks 0, 1, 3, 6, and 10. Stimulated cells were fixed, permeabilized and stained with αIFNγ-FITC, αCD69-PE, αCD8-PerCP and αCD4-APC. The percent of CD4+ and CD8+ T cells that are IFNγ and CD69 double positive are represented for each antigen. Cells were gated by forward and side scatter for lymphocytes and positively for CD8 or CD4. Arrows represent when the patient was given each vaccination: week 0, 3, 6 and 9. The x-axis represents week of analysis and y-axis represents percent CD4+ or CD8+ cells that are CD69+IFNγ+ in response to each antigen.

As further confirmation of the HER2 specificity of the immune response, we analyzed patient PBMC by ELISpot and intracellular cytokine staining. ELISpot analysis was performed on the PBMCs from each patient prior to vaccinations (pre), following vaccinations (post), and at long term follow up (up to 5 years following immunizations) (Table 2). We initially reported our results in three patients for immune response during the immunizations [19]. We now report results for all patients with longer follow-up (up to 5 years). As demonstrated in Table 2, 3 of 3 patients receiving immature DC, and 2 of 3 receiving matured DC developed a positive or increased response to ICD protein as demonstrated by ELISpot results. Out of these 5 patients, 3 had an immune response to Her2-ICD up to 5 years following the DC vaccinations. Interestingly, these results indicate the Her2-ICD specific T cell response was not always greatest at the conclusion of the immunizations. Her2-I-01 and DC-ICD-MAT-03 also had Her2-ECD specific responses up to 5 years following immunizations. The patient receiving FLT3-Ligand mobilized and matured DC did not activate ICD-specific T cells but had in increase in ECD specific T cells. In patient Her2-I-01, we confirmed that the Her2-ICD immune response occurred among both CD4 and CD8+ T cells by intracellular IFNγ production (Figure 1).

**Table 3:** Patient ELISpot response

**Patient**	**Antigen**	**Pre**	**Post**	**Follow up 1**	**Follow up 2**
**Her2-I-01**	ICD	-	-	+ (150)	+ (23)
	ECD	-	+ (23)	+ (114)	+ (24)
**Her2-I-02**	ICD	-	+ (22)	-	N/A
	ECD	-	N/A	-	N/A
**Her2-I-03**	ICD	-	-	-	+ (23)
	ECD	+ (49)	-	-	+ (23)
**DC-ICD-MAT-01**	ICD	-	+ (18)	N/A	-
	ECD	-	-	N/A	-
**DC-ICD-MAT-02**	ICD	N/A	-	-	-
	ECD	N/A	-	-	-
**DC-ICD-MAT-03**	ICD	-	N/A	N/A	+ (77)
	ECD	-	N/A	N/A	+ (129)
**FL-DC-MAT-01**	ICD	-	-	-	-
	ECD	+ (46)	-	-	+ (84)

A positive ELISpot result is represented by + and the number of spots per 100,000 PBMC in parentheses. A positive (+) result is defined as the number of cells secreting IFNγ in response to HERr2-ICD or ECD that is at least 10 more than the number of cells secreting IFNγ in response to media alone (control cells) and which is statistically greater than the control cells with p < 0.05.

Each antigen was tested in replicates of 6 for each time point.

Pre represents ELISpot results prior to vaccination, post represents results during or following the vaccination series, and the follow up timepoints represent up to 5 years following the study.

**Figure 2:**
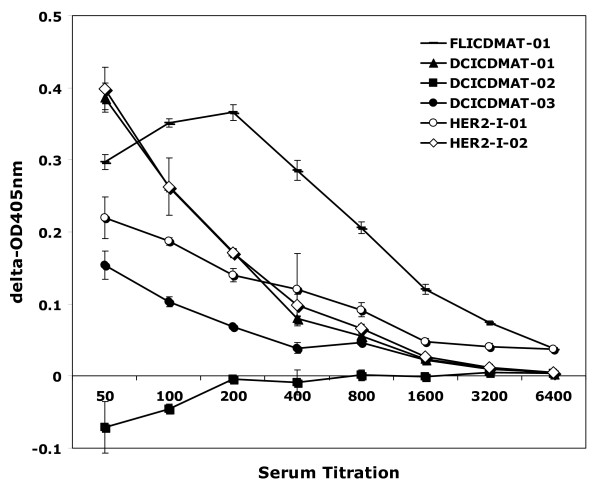
**Antibody response to HER2-ICD protein detected by ELISA**. High binding 96-well microtiter plates were coated with HER2-ICD protein (10 μg/ml, 200 μl/well) for overnight at 4°C. After washing, and blocking with 1% BSA in PBS for 2 hours, serially diluted patients' sera (200 μl/well) were added to the wells in triplicate and incubated overnight at 4°C. Control serum from a normal donor was used as a negative control. Alkaline phosphatase conjugated anti-human IgG was added to the plate after washing, and color was developed using p-nitrophenyl phosphate. Absorbance at 405 nm was measured and the differences from the control serum at each dilution was plotted. The data represents the mean absorbance for each serum dilution ± standard deviation for each patient depicted.

Although T cell responses were the initial primary immune response measured, when the subjects were evaluated at more than 5 years of follow-up, we analyzed antibody responses specific for ICD by ELISA. As demonstrated in figure 2, five of 6 evaluable patients had detectable antibody responses against the ICD with titers exceeding 1:200. There was no obvious correlation with the T cell responses, although it is of interest that the patient who received the Flt3-Ligand mobilized DC developed the highest titer of anti-ICD antibody response, but had minimal detectable T cell secretion of interferon-gamma, suggesting a possible skewing of the T cell repertoire to a Th2 response.

### Clinical Observations

No acute toxicities were noted during or following any of the four injections. No patient demonstrated an autoimmune response, and no cardiotoxicity, defined as decreased ejection fraction below normal(< 50% at our institution or decrease of > 10% from their baseline), was observed on repeat MUGA scans or echocardiograms among the six patients for whom follow-up evaluations were available. In the remaining patient no cardiac events have been reported, and the patient remains fully functional and active. The only patient-reported toxicities were related to prior chemotherapy and/or surgery (e.g., neuropathy and lymphedema, respectively).

**Figure 3:**
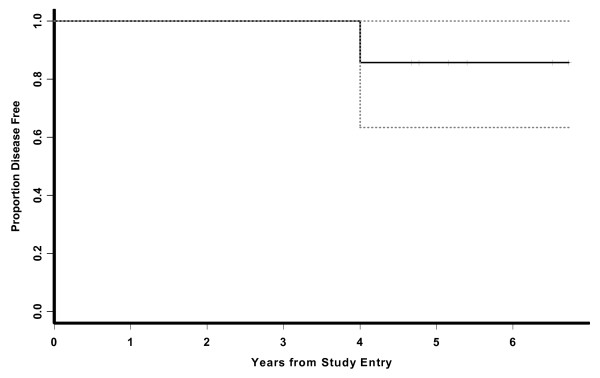
Kaplan-Meier Disease-Free Survival Estimate and 95% confidence bounds (n = 7).

Only one of seven patients experienced disease recurrence; she developed a single HER2+ pulmonary lesion nearly 4 years after completing all immunizations and is currently disease-free 9 months post-resection and chemotherapy (Figure 3). With a median follow-up of 5.2 years (range:4.6–6.7 years), the DFS rate is 86% and the overall survival is 100%. The 95% exact binomial confidence interval for survival at 4.5 years post-immunization is (59%, 100%).

## Discussion

Patients with resectable, HER2-expressing breast cancer have a substantial (30% to > 50%) risk for recurrence with conventional therapy. Recently, a combined analysis of 1672 HER2+ patients who were treated with doxorubicin, cyclophosphamide and paclitaxel plus one year of trastuzumab demonstrated a 52% reduction in the unadjusted risk for disease recurrence and a 33% reduction in the unadjusted risk for mortality compared to those who did not receive trastuzumab. Four-year disease-free survival (DFS) and OS rates were 85.3% and 91%, respectively [2]. This required a year of therapy, and as a passive approach, it only provides activity while the drug persists. We hypothesized that a cancer vaccine strategy would provide prolonged efficacy due to T cell immunity, and this report describes the high rate of long term DFS of seven patients with high-risk, resected, HER2 overexpressing breast cancer after immunization with dendritic cell based vaccines loaded with the ICD protein.

Our study is noteworthy for several reasons. First, we were able to generate DCs from PBMCs in all patients despite significant prior chemotherapy. This demonstrates the feasibility of this approach in the post-surgery/post-chemotherapy setting. Notwithstanding, two patients in the group who received matured DCs did not receive all 4 immunizations because the manipulations required to mature and load the DCs with antigen produced significant effects on the number and viability of cells. Three different DC-based preparations were utilized among the seven patients because we were intending to test each for safety in a pilot fashion as we continued to develop more convenient and effective DC vaccines. We realize that the number of different preparations and prior therapies of the patients may limit the ability to make conclusions, but nonetheless, all the patients did have in common immunization with ICD and ECD and prolonged relapse-free survival and 6 of the 7 patients showed development of HER2 specific immune responses. Unfortunately, our ability to continue the study was ultimately limited by the unavailability of clinical grade ICD protein and FLT3-Ligand. The generation of protein-based vaccines is complicated by the complexity of producing stable proteins. Furthermore, there was no evidence that the manipulations required to mature the DC resulted in greater immunogenicity. Neither did the Flt3-ligand mobilized DC approach seem to improve the T cell responses against the ICD protein, although it was associated with the highest antibody titer at 5 years of follow-up. Similarly, Disis and colleagues [8] reported that Flt3-Ligand did not activate a HER2-specific proliferative response, although they could detect Her2-specific T cells responses by ELISPOT. In order to improve upon the immunogenicity of the vaccines used in the current study, we have other strategies in development including heterologous prime-boost strategies using viral vectors and DC-based vaccines modified with viral vectors encoding HER2.

Second, we observed vaccine specific immune responses by DTH in six of seven patients with HER2 ICD protein administered with an *ex vivo *generated DC. We confirmed that these were HER2 ICD-specific T cell responses in five (two in the mature DC group and three in the immature DC group) of seven immunized patients. At more than 5 years of follow-up, ICD and ECD-specific immune responses were detected by ELISPOT and in some cases, the results were higher than during the immunizations providing tantalizing evidence that the peak response may occur significantly after concluding vaccine therapy. Similar observations have been made in other HER2 vaccine studies. Disis and colleagues demonstrated anti-HER2, T-cell immunity persisting for at least 1 year among 38% of patients responding to a vaccine derived from potential T-helper epitopes of the HER2/*neu *protein admixed with granulocyte-macrophage colony-stimulating factor (GM-CSF) [7]. In another study by the same group, over half of patients (n = 29) enrolled in a dose-escalation study retained HER2/*neu *specific T-cell immunity after treatment with HER2ICD protein plus GM-CSF vaccine 9 to 12 months after immunization had ended [11]. Interestingly, the rate of immune response seemed to decrease slightly with increasing dose of vaccine (100% for low dose, 90% for intermediate dose, and 78% for high dose). In some cases, there was a rebound of the T cell response 2–3 months (or later) after the immunizations were completed. Although efficacy was not reported in this phase I trial, patients with stage II, III and IV HER2 overexpressing breast cancer were enrolled. Also, although we did not have serum remaining from prior to immunization to detect antibody responses, we were encouraged to find detectable antibody responses specific for the ICD protein in 5 of 6 patients at 5 years of follow-up. Although existent antibody responses against HER2 have been detected in non-immunized patients [20,21], the fact that the Ab response was against the intracellular domain (ICD) suggests that this response could have been activated by the vaccine.

Third, although the number of patients was limited by the eventual unavailability of the ICD protein and FLT3-Ligand, the fact that all seven (100%) are alive and currently disease-free for up to 6 years following the final vaccination is noteworthy in our opinion. Disis [11] does not report the percentage of patients alive and disease free, but does mention that 2/29 patients relapsed before completing the vaccine series (all were in the low dose cohort). Although two of our patients had also received trastuzumab, one had received it for only 12 weeks; the other has continued on trastuzumab for more than 4 years after completing the immunizations. We are aware that some patients received adjuvant hormonal therapy which could have influenced relapse as well. Larger cohorts of patients must be studied to confirm these results.

Fourth, the immunizations were well tolerated with only grade 1 toxicities reported. Cardiotoxicity due to the immunizations was not reported, despite the fact that two patients had also received trastuzumab and the majority had received prior anthracyclines.

## Conclusion

In this small pilot study, we observed that DC vaccines loaded with HER2 ICD protein are well tolerated, induce DTH, T cell, and antibody responses, and are associated with long term-disease-free survival. It is noteworthy to compare these results with another HER2 directed therapy in which the concept of prolonged immunity and its application to the adjuvant treatment setting was recently explored in patients with resected node positive and node negative breast cancer [13]. A vaccine consisting of a peptide derived from the extracellular domain of HER2 (E75 peptide (HER2 369–377)) mixed with GM-CSF was administered at various doses and schedules. All patients demonstrated in vivo DTH responses and in vitro immunologic responses following vaccination. The recurrence rate for the vaccinated patients was 5.6% compared to 14.8% for an observational group of patients at a median of 24 months. These observations are promising and support further study of anti-HER2 vaccine therapy after adjuvant treatment of high-risk, HER2 overexpressing breast cancer. Because trastuzumab is now a standard treatment administered to women with HER2 overexpressing breast cancer as part of adjuvant therapy, and because the safety of trastuzumab plus HER2 targeting vaccines has been preliminarily demonstrated [22], future studies will assess the combination of DC-based HER2 targeting vaccines with trastuzumab.

## Competing interests

The author(s) declare that they have no competing interests.

## Authors' contributions

MM and KB carried out clinical trial research.

AH and TC participated in the writing of the manuscript.

DN performed statistical analysis.

GD provided expert opinion for the study.

CA and PM were involved in consultation for the trial.

HL was the initiator of the study.

TO developed, performed, and interpretted ELISA assays.
